# Unconscious response inhibition differences between table tennis athletes and non-athletes

**DOI:** 10.7717/peerj.5548

**Published:** 2018-09-07

**Authors:** Yihong You, Yiming Ma, Zhiguang Ji, Fanying Meng, Anmin Li, Chunhua Zhang

**Affiliations:** 1 School of Kinesiology, Shanghai University of Sport, Shanghai, China; 2 Faculty of Behaviour and Movements Science, VU University Amsterdam, Amsterdam, The Netherlands

**Keywords:** Unconscious response inhibition, Table tennis athletes, Feedforward sweep, Recurrent processing

## Abstract

**Background:**

Response inhibition is associated with successful sporting performance. However, research on response inhibition in athletes from open-skill sports has mainly focused on a consciously triggered variety; little is known about open-skill athletes’ response inhibition elicited by unconscious stimuli.

**Methods:**

Here, we explored unconscious response inhibition differences between table tennis athletes (*n* = 20) and non-athletes (*n* = 19) using the masked go/no-go task and event-related potentials technique (ERPs).

**Results:**

At the behavioral level, table tennis athletes displayed shorter go-response times (RTs) than non-athletes in the conscious condition. Furthermore, table tennis athletes exhibited longer response time–slowing (RT-slowing) than non-athletes in the unconscious condition. At the neural level, table tennis athletes displayed shorter event-related potential N2 component latencies than non-athletes for all conditions. More importantly, athletes displayed larger no-go event-related potential P3 component amplitudes than non-athletes at both the conscious and unconscious levels.

**Discussion:**

The present study results suggested that table tennis athletes have superior conscious and unconscious response inhibition compared to non-athletes.

## Introduction

Response inhibition is an executive function that enables suppression of no-longer appropriate or inappropriate behavioral responses in a given context (Liddle, Kiehl & Smith, 2001). Previous studies have shown that response inhibition is associated with successful sporting performance (e.g., Vestberg et al., 2012), albeit that the strength of the association depends on sport-specific cognitive and motor demands. In this respect, two types of skills can be distinguished in sports: open skills, in which players have to react in a dynamically changing, unpredictable environment and skills are often externally-paced (e.g., table tennis, tennis, and fencing); and closed skills, in which the sporting environment is relatively constant, predictable, and skills are typically self-paced (e.g., swimming) (Wang et al., 2013). It is well established that athletes from open skill sports display superior response inhibition compared to non-athletes and athletes from closed skill sports. Likely, the superiority in response inhibition is a result of the long-term response inhibition experience (Chan et al., 2011; Di Russo et al., 2006; Nakamoto & Mori, 2008b; Wang et al., 2013). However, this inference is mainly based on studies that examined response inhibition elicited by stimuli or events that are consciously perceived, which we define here as conscious response inhibition. By contrast, response inhibition elicited by stimuli or events of which athletes are not conscious aware (Van Gaal et al., 2011), which we define as unconscious response inhibition, have largely been ignored. Hence, the focus of the current research is to assess whether athletes from open skill sports also show superior unconscious response inhibition compared to non-athletes.

Conscious response inhibition in athletes from open skill sports have been studied using the traditional go/no-go task and event-related potentials technique (ERPs). The majority of these studies have shown that athletes from open skill sports display superior conscious response inhibition compared to non-athletes (e.g.,  Nakamoto & Mori, 2008b; Wang et al., 2013). The go/no-go task requires participants to respond as quickly as possible to a go stimulus, but to suppress a response for a no-go stimulus. In addition, ERPs provide a high temporal resolution measure of brain activity related to response inhibition. Two ERP-components are typically associated with successful conscious response inhibition. The frontocentral no-go N2 component is elicited in no-go trials, comprising a negative shift between 200 ms and 300 ms. It reflects conflict monitoring processes in the early stage of conscious response inhibition (Donkers & Van Boxtel, 2004). The no-go P3 component is also elicited in no-go trials but refers to a positive wave that peaks between 300 ms and 600 ms. This reflects a later stage of the conscious response inhibition (Bokura, Yamaguchi & Kobayashi, 2001). In addition, to the conscious response inhibition itself, the no-go P3 may also reflect evaluation of the conscious response inhibition (Beste et al., 2009; Beste et al., 2010; Roche et al., 2005). The two classic ERP components are larger in the no-go trials than in the go trials.

For example, Nakamoto & Mori (2008a) presented baseball players with a go/no-go task. The baseball players displayed shorter response times (RTs) and a larger no-go P3 amplitude (Fz) in spatial conditions with baseball batting-specific stimulus–response mapping (i.e., participants were instructed to respond as quickly and accurately as possible when the color of either of the two center squares changed from black to green; participants were instructed to inhibit this response when the color of either of the two non-centered squares changed) in comparison to spatial conditions without baseball batting-specific stimulus–response mapping. This difference was not observed in non-athletes. This indicates that baseball athletes have enhanced conscious response inhibition for batting-specific stimulus–response mappings only. However, other research has suggested that superiority in conscious response inhibition may not be limited to sports-specific stimulus–response mappings (Wang et al., 2013; Vestberg et al., 2012). For example, Wang et al. (2013) used a general stop-signal task (i.e., without a sports-specific stimulus–response mappings) and compared conscious response control in athletes from open skill sports (i.e., tennis) with that in athletes from closed skill sports (i.e., swimming) and sedentary students. Tennis players showed shorter stop-signal reaction times than both the swimmers and non-athlete students, suggesting that athletes from open skill sports have better conscious response inhibition than other persons, even in tasks that are not sports-specific.

In contrast to conscious response inhibition, much less is known about the degree to which athletes from open skill sports distinguish themselves with respect to unconscious response inhibition. Various interactive sports (e.g., returning a tennis serve, batting in baseball and cricket, stopping a soccer penalty kick) involve rapid motor responses under very high time pressure (Kibele, 2006). Researchers have argued that relying solely on explicit, conscious evaluations of the situation would require too much time for a timely response (Güldenpenning et al., 2015; Van der Kamp et al., 2008; Williams & Ward, 2007). Importantly, studies have suggested that athletes from open skill sports display better unconscious information processing than non-athletes in coping with high time pressure situations (Güldenpenning et al., 2015; Güldenpenning et al., 2011; Koester, Schack & Güldenpenning, 2017). For example, one study used a masked priming tasks to explore the differences in unconscious perception and unconscious motor responses between athletes of martial arts and novice participants. Athletes of martial arts and novice participants were found to have similar ability to unconsciously distinguish feint and non-feint actions by opponents but athletes of martial arts were faster in initiating a motor response (Güldenpenning et al., 2015), underlining that also unconscious information processing is vital to the performances of athletes from open skill sports. Here, we explore whether athletes from open skill sports also show superior unconscious response inhibition compared to non-athletes.

Researchers have recently demonstrated that response inhibition can also occur unconsciously, without the participant being aware of the stimulus (Chiu & Aron, 2014; Lau & Passingham, 2007; Van Gaal et al., 2011; Jiang et al., 2014). To this end, functional magnetic resonance imaging and a new version of the go/no-go task, involving masking, was used (Van Gaal et al., 2010). This masked go/no-go task included both weakly masked (i.e., conscious) and strongly masked (i.e., unconscious) go/no-go trials. Participants were asked to respond to a metacontrast annulus as fast as possible but to withhold their response when they perceived a no-go stimulus preceding the metacontrast annulus. However, when a go stimulus preceded the metacontrast annulus, they were instructed to also respond as quickly as possible. By manipulating the stimulus onset asynchrony between the go/no-go stimulus and the metacontrast annulus, the go/no-go stimulus was either perceived consciously or unconsciously. Thus, in the weakly masked condition, participants would respond like the traditional go/no-go task because they consciously perceive the go/no-go stimulus. However, in the strongly masked condition, in which the no-go stimulus is not consciously perceived, a go response is expected (i.e., providing a direct RT measure for the unconscious no-go trials). An index of unconscious response inhibition is obtained by taking the RTs for strongly masked no-go trials minus the RTs for strongly masked go trials (i.e., RT-slowing). Van Gaal and colleagues showed that the strongly masked no-go stimulus did result in a slower response time and even occasionally triggered response inhibition. Moreover, the strongly masked no-go stimulus was also accompanied by activation of the frontoparietal “inhibition network”, particularly in the inferior frontal cortex and the presupplementary motor area. Further studies reported similar behavioral results (e.g., Van Gaal et al., 2011) and confirmed that unconscious no-go stimulus triggered the no-go N2 and no-go P3 ERP-components as was earlier established for conscious response inhibition (Van Gaal et al., 2008; Wokke et al., 2011).

Even though unconscious and conscious no-go stimuli have been shown to activate the prefrontal network and elicit the same ERP-components, unconscious no-go stimuli fail to elicit the comparably large, strong, and distributed pattern of activation that is observed for conscious no-go stimuli (Van Gaal et al., 2011; Van Gaal et al., 2008; Van Gaal et al., 2010). Hence, to clarify the relationship between conscious and unconscious response inhibition, it is proposed that feedforward sweep and recurrent processing have different roles in conscious and unconscious perception (Van Gaal et al., 2010). Feedforward sweep refers to the earliest activation of cells in successive areas of the cortical hierarchy. Recurrent processing involves an interaction between higher- and lower-level brain areas that enables information to be broadcasted across the brain and maintained across time (Klink et al., 2015; Lamme, 2006). Van Gaal and colleagues proposed that the early feedforward sweep may remain unconscious, whereas recurrent processing triggers awareness of a stimulus. They claimed that unconscious no-go stimuli can evoke feedforward activation of the same cortical areas as conscious no-go stimuli do, including areas further in the cortical hierarchy, thus activating the same prefrontal network and eliciting the same ERP-components. However, with unconscious no-go stimuli feedforward activation may die out quickly because it is not supported by recurrent processing. It is for this reason that unconscious no-go stimuli fail to elicit a comparably large, strong and distributed pattern of activation as conscious no-go stimuli.

The present study examines differences in unconscious response inhibition between athletes from open skill sports and non-athletes. Following the proposal that conscious and unconscious response inhibition share the same initial information processing stage (i.e., feedforward sweep), it was hypothesized that, depending on the degree to which the athletes’ superior conscious response inhibition arises within this initial information processing stage, similar advantages in unconscious response inhibition would be present in athletes from open skill sports. To examine this, athletes from table tennis, a classic open skill sport, were recruited for the present study. Table tennis is an interactive racket sport that is characterized by extremely high time pressure. It requires superior motor control, fast interceptive actions, hand–eye coordination, and a high perception–action demand (Wang et al., 2013). Importantly, table tennis athletes have been shown to exhibit superior conscious response inhibition (Lenoir et al., 2000). We employed a masked go/no-go task with ERP measures to compare conscious and unconscious response inhibition in table tennis athletes and non-athletes. We expected more accurate inhibition, shorter response times and longer RT-slowing, and larger no-go N2- and P3-components amplitudes in the table tennis athletes than in the non-athletes for both the weakly and strongly masked go/no-go conditions.

## Methods and Materials

### Ethical approval

This study received approval from the ethics committee of Shanghai University of Sport (No. 2017033). All participants provided written informed consent before participating, and each received 50 RMB for their participation in this study.

### Participants

Participant demographic characteristics and level of physical activity are shown in Table 1. Power analysis (G*Power3.1, *α* = 0.05, power = 0.80, effect size = 0.25) showed that a minimum 34 volunteers needed to participate. After recruitment, 39 volunteers were divided into two groups: (1) 19 non-athletes (mean [SE] age, 21.26 [0.56] years); and (2) 20 table tennis athletes (mean [SE] age, 20.65 [0.39] years). All athletes were qualified for at least the national second level in China before they attended university and had at least 8 years of table tennis experience and practice. On average, they had 10.68 years of table tennis experience and practice. Non-athletes occasionally participated in some sports activities, but none were active at a competitive level. All participants were recruited from Shanghai University of Sport. All participants had normal or corrected-to-normal vision and were right-handed; handedness was self-reported. Because it has been reported that body mass index (BMI) is negatively associated with conscious response inhibition (Batterink, Yokum & Stice, 2010), also BMI was measured across groups prior to the experiments. A previous study showed that physical activity is positively correlated with executive control (Kamijo & Takeda, 2009). Hence, we used a 7-day physical activity recall questionnaire (IPAQ) to assess the participants’ physical activity. Four levels of physical activity were classified using the IPAQ, and each intensity level was indicated by a metabolic equivalent (MET) as follows: high activity = 8 METs; moderate activity = 4 METs; walking = 3.3 METs; and sitting = 1 MET. There were no significant differences in age, weight, height, BMI, or level of physical activity between the two groups (Table 1).

**Table 1:** Participant demographic characteristics and physical activity.

**Variable**	**Non-athletes,mean (SE)(*n* = 19)**	**Table tennis athletes,mean (SE)(*n* = 20)**	***t*(37)**
Age (years)	21.26 (0.56)	20.65 (0.39)	0.91
Female (No.)	10	9	
Height (cm)	169.05 (2.16)	170.80 (1.91)	−0.61
Weight (kg)	63.50 (2.42)	61.93 (2.02)	0.50
BMI (kg/m^2^)	22.09 (0.45)	21.14 (0.43)	1.53
Physical activity (METs-min/w)	3,629.11 (717.62)	4,186.70 (605.81)	−0.60
Table tennis experience (years)	NA	10.68 (0.32)	NA

**Notes.**

BMI body mass index METs metabolic equivalents NA not applicable

### Masked go/no-go task

**Figure 1:**
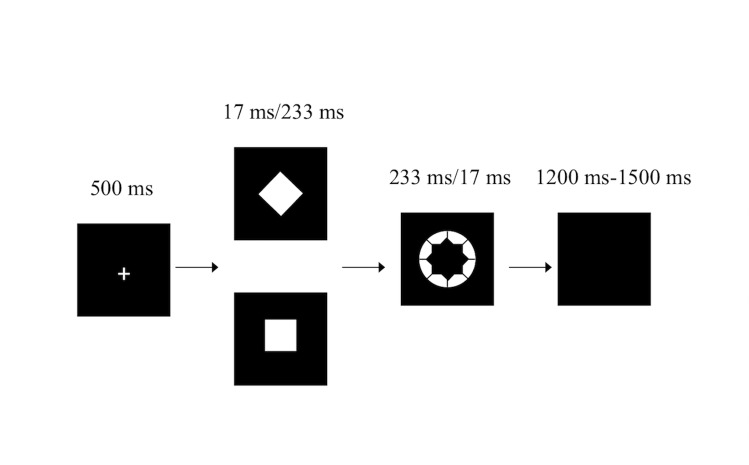
The masked go/no-go task.

The masked go/no-go task has been described previously (Van Gaal et al., 2010; Xu et al., 2015) (Fig. 1). Briefly, white stimuli were presented in the center of a screen against a black background on a 19-inch DELL TFT computer with a refresh rate of 60 Hz. The participants were instructed to sit 60 cm from the front of the computer. Participants were instructed to respond to the white annulus (visual angle 0.8°) as quickly as possible by pressing the “2” key on a standard keyboard with the right index finger but to withhold their response when a white diamond (the no-go stimulus, visual angle 0.47° × 0.47°) preceded the white annulus. However, participants were instructed to respond as quickly as possible by pressing the “2” key when a white square (the go stimulus, the same diamond but tilted by 45°) preceded the white annulus.

In the weakly masked condition, the white annulus was ineffective in masking the white square/diamond, since the square/diamond was presented for 233 ms and the white annulus was presented for 17 ms. In the strongly masked condition, the square/diamond was presented for 17 ms and was followed by the white annulus (233 ms). The white annulus functioned as a metacontrast mask, which has been proven to strongly reduce stimulus visibility (Breitmeyer, 1984). The combination of the white annulus and the fairly short stimulus onset asynchrony of the square/diamond prohibited participants from perceiving the square/diamond consciously. Thus, in the weakly masked condition, participants were expected to respond as specified by the go and no-go stimuli because both stimuli are consciously perceived, while participants in the strongly masked condition were expected to almost always make a go response (i.e., also for no-go stimuli) since the no-go stimulus was not consciously perceived.

The weakly masked and strongly masked go and no-go trials were randomly mixed in blocks. The task consisted of four blocks, with each block containing 120 trials for a total of 480 trials (30 trials of each trial type per block). The stimulus used as the go/no-go stimulus (square or diamond) was counterbalanced across participants (Van Gaal et al., 2010). Participants frequently pressed the keyboard but also infrequently inhibited this action, making this a go/no-go task instead of a simple detection task.

The participants completed a practice block of 16 trials (four trials for each trial type) before the actual experiment began. This approach was used to ensure that all participants understood the task instructions.

### Awareness task

To ensure that none of the participants perceived the go/no-go stimulus (square or diamond) in the strongly masked condition, a standard test was performed after the masked go/no-go task to assess participants’ unconsciousness perception (Van Gaal et al., 2011; Xu et al., 2015). The stimuli and trial types in this test were the same as in the masked go/no-go task. However, the procedure differed from the masked go/no-go task as the participants were informed about the strongly masked condition beforehand. In the awareness task, the participants were instructed to press the “V” key when they perceived a square before the annulus and to press the “N” key when they perceived a diamond before the annulus. Furthermore, they were informed that response accuracy was important in this task, whereas the response time was not important. This task comprised only one block of 30 trials for each trial type.

### Overall procedure

After providing informed consent, the participants joined the three phases of the experiment. In the first phase, participants completed the physical activity recall questionnaire and provided their basic demographic information. In the second phase, participants performed the masked go/no-go task, with also EEG data being recorded. In the third phase, the participants performed the awareness task. The mean duration of the entire experiment was approximately 1.5 h (50 min to set up the EEG, and 40 min for the participants to complete the assigned task).

### Behavioral analysis

All demographic variables were analyzed using independent two-tailed, unpaired *t* tests (see Table 1).

A binominal test was used to analyze data obtained from the awareness task (Wokke et al., 2011). Participants whose percentages of correct responses were significantly above the chance level in the strongly masked condition were to be removed, because it could not be ensured that they truly could not perceive the strongly masked go/no-go stimuli. A one-sample *t* test was separately performed on the sensitivity index (*d*′) (tested against 0) for each group for the strongly masked condition (Xu et al., 2015). In addition, the group differences for the *d*′ scores were analyzed using independent *t* tests.

In the masked go/no-go task, reaction times less than 100 ms and greater than 1,000 ms were excluded from all analyses (Wokke et al., 2011). In the weakly masked condition, group differences were submitted to an independent *t* test to analyze the go-RTs, and a two-way repeated analysis of variance (ANOVA; 2 [group: table tennis athlete, non-athlete] × 2 [trial type: go, no-go]) was used to analyze accuracy. A two-way repeated ANOVA (2 [group: table tennis athlete, non-athlete] × 2 [trial type: go, no-go]) was used to analyze the RTs and inhibition rate for the strongly masked condition. The differences in RT-slowing (i.e., the strongly masked no-go RTs minus the strongly masked go RTs) between the two groups were analyzed using independent *t* tests.

### EEG measurements and analyses

The EEG activity was recorded with a low-pass filter of 100 Hz and sampled at 500 Hz using a Brain Vision system with 64 electrodes referenced to FCz. Horizontal eye movements were recorded from an electrode placed on the outer canthus of the right eye, and vertical eye movements were recorded with an electrode placed below the left eye. Electrode impedance was below 10 kΩ. After acquisition, the EEG data were referenced to the average of the left and right mastoid processes. Eye movement correction was applied based on independent component analysis, and EEG data were filtered using a low-pass filter of 30 Hz. Artifact rejection was applied by semi-automatically removing segments outside the range of ±80 µV. Finally, ERPs were averaged in epochs, which lasted 900 ms and began 200 ms before the go/no-go stimulus onset. In the weakly masked condition, only correct responses were included in the analysis; however, we analyzed go response in the strongly masked condition because participants in the strongly masked condition were expected to almost always make a go response. All preprocessing steps were conducted using Brain Vision Analyzer (version 2.1).

Previous studies have shown that there are two main ERP-components related to conscious response inhibition (Bokura, Yamaguchi & Kobayashi, 2001): the no-go N2, and the no-go P3 components. Because midline electrodes elicit the most obvious N2 and P3 components (Ma et al., 2015) and previous studies have shown that the no-go N2 and no-go P3 components are maximal in the frontocentral areas (Bokura, Yamaguchi & Kobayashi, 2001; Eimer, 1993), we selected three frontocentral electrodes in the midline position (Fz, FCz, and Cz) to analyze the amplitude and latency values of the N2 and P3 components. Using the grand average in weakly and strongly masked condition, we analyzed the maximum negative amplitude in the interval from 200–350 ms for the N2 component in both conditions. For the P3 component, we analyzed the maximum positive amplitude in the interval from 350–550 ms in the weakly masked condition, and the maximum positive amplitude in the interval from 440–550 ms in the strongly masked condition. For each participant, the peak amplitude and peak latency measures were semi-automatically detected in a given time window.

A three-way repeated ANOVA (2 [group: table tennis athlete, non-athlete] × 2 [trial type: go, no-go] × 3 [electrode site: Fz, FCz, and Cz]) was performed to analyze the peak amplitude and peak latency values of the N2 and P3 components in each condition. A Greenhouse-Geisser correction was used to compensate for sphericity violations. Least significant difference tests were used to explore multiple comparisons, and a simple effects analysis was used to explore interaction effects. Values of *p* < 0.05 were considered statistically significant.

## Results

(The raw data is shared as a Supplemental File and at Figshare: https://figshare.com/s/40d9d25101fd87035cc7).

Two participants were excluded from the non-athlete group and four participants were excluded from the table tennis athlete group because their accuracies were above the chance level in the strongly masked condition in the awareness task. The subsequent analyses included only the remaining subjects (*n* = 33).

### Awareness task

For the *d*′ scores, the analysis showed that there was no significant difference from 0 in either group (table tennis athletes: mean [SE], −0.25 [0.27]; non-athletes: mean [SE], 0.12, [0.15]), which indicates that all participants were unable to consciously perceive the go/no-go stimulus in the strongly masked condition. Furthermore, there was no significant difference between the table tennis athlete group and non-athlete group (*t* (31) = 1.23, *p* = 0. 229), indicating that the two groups did not differ significantly in the perception of the strongly masked go/no-go stimulus.

### Masked go/no-go task

#### Weakly masked condition

There was a significant group effect for go-RTs (*t* (31) = 2.87, *p* < 0.05, *d* = 1.01), indicating that the table tennis athletes had faster go-RTs than the non-athletes; however, no differences were identified between the groups for either go accuracy (*F*_(1,31)_ = 1.50, *p* > 0.05) or no-go accuracy (*F*_(1,31)_ = 0.03, *p* > 0.05) (Table 2).

#### Strongly masked condition

**Figure 2:**
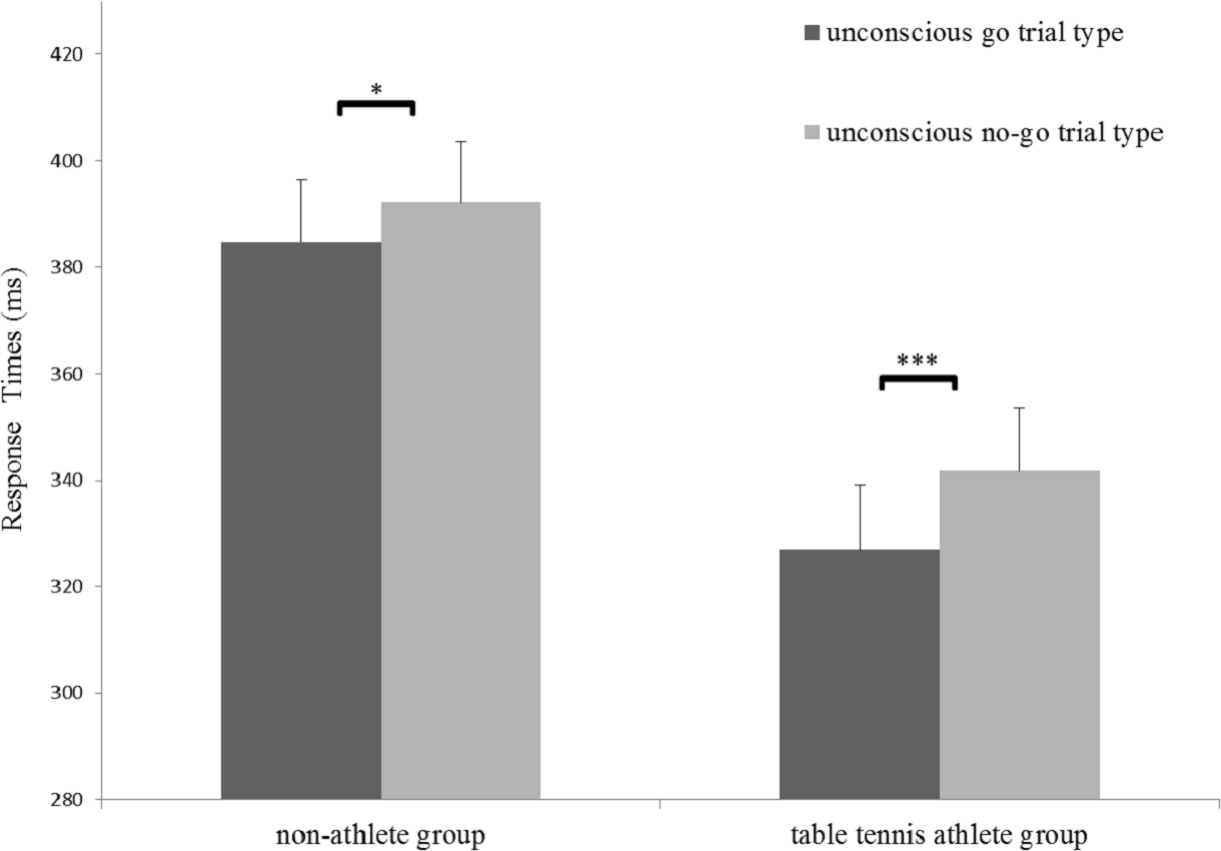
Response times in the two groups for the strongly masked condition. *p < 0.05; ***p < 0.001.

A significant main effect of trial type for RTs was identified (*F*_(1,31)_ = 45.73, *p* < 0.001, *η*^2^ = 0.56), indicating that RTs for the no-go trial (mean [SE], 366.94 [8.26] ms) was longer than that in for the go trials (mean [SE], 355.87 [8.47] ms). In addition, a significant main effect of group for RTs (*F*_(1,31)_ = 10.60, *p* < 0.05, *η*^2^ = 0.26) and a significant interaction between group and trial type was identified (*F*_(1,31)_ = 5.05, *p* < 0.05, *η*^2^ = 0.06). A simple effects analysis showed that the RTs in the no-go trial were significantly longer than those in the go trial for both groups (non-athlete: *F*_(1,31)_ = 10.51, *p* < 0.05, *η*^2^ = 0.25; table tennis athlete group: *F*_(1,31)_ = 39.39, *p* < 0.001, *η*^2^= 0.56) (Fig. 2). More importantly, there was a significant difference in RT-slowing (*t* (31) = − 2.25, *p* < 0.05, *d*= 0.78) between the groups, indicating that RT-slowing in the table tennis athletes was greater than that in non-athletes. No significant effects for inhibition rate were identified (Table 2).

**Table 2:** Behavioral results of the weakly (conscious) and strongly (unconscious) masked go/no-go task for the two groups.

**Variable**	**Non-athletes,mean (SE)(*n* = 17)**	**Table tennis athletes,mean (SE)(*n* = 16)**
Conscious go-RTs (ms)	416.86 (17.51)	352.90 (13.42)^*^
Conscious go-ACC (%)	97.91 (0.67)	99.08 (0.69)
Conscious no-go-ACC (%)	71.84 (4.19)	72.83 (4.32)
Unconscious go-RTs (ms)	384.81 (11.80)	326.92 (12.16)^*^
Unconscious go-IR (%)	1.87 (0.69)	1.00 (0.71)
Unconscious no-go-RTs (ms)	392.21 (11.50)	341.68 (11.86)^*^
Unconscious no-go-IR (%)	2.00 (0.82)	1.79 (0.85)
RT-slowing (ms)	7.40 (1.90)	14.76 (2.70)^*^

**Notes.**

ACC accuracy RTs response times IR inhibition rate

* *p* < 0.05 between the two groups.

### ERP results

#### Weakly masked condition

**Figure 3:**
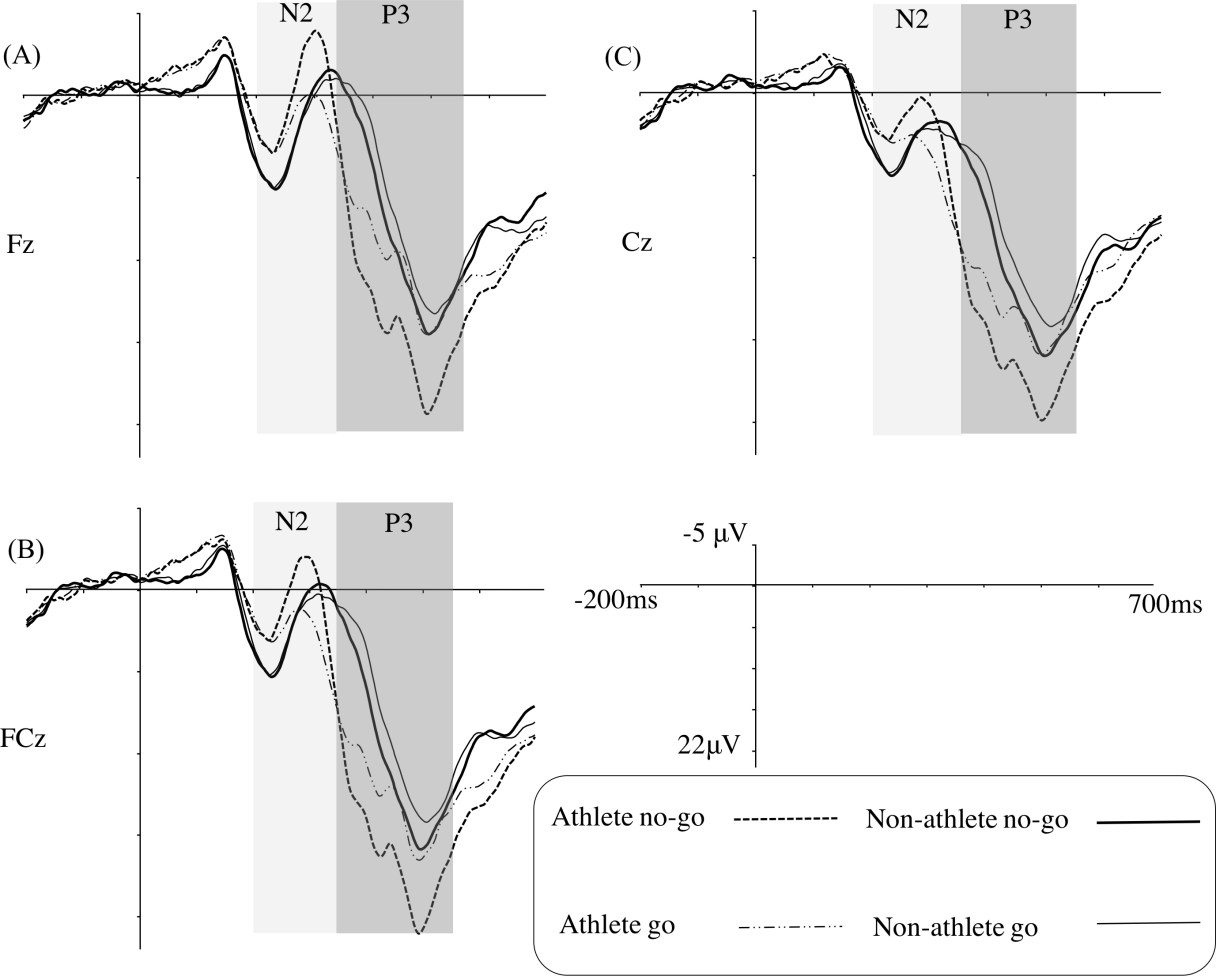
Grand average ERPs in the weakly masked condition for the go and no-go trials in the table tennis athlete and non-athlete groups for each electrode site (A–C represents Fz, FCz, and Cz, respectively).

Figure 3 illustrates the grand average ERPs at Fz, FCz, and Cz for each group in the weakly masked condition, and Table 3 gives the results of the corresponding statistical analyses.

**Table 3:** Statistical analysis results of the electrophysiological data in the weakly masked condition.

**Variable**	**Component**	**Effect**	***F***	***p***	*η*^2^
Amplitude	N2	Trial type	14.47	=0.001	0.28
		Electrode site	59.66	<0.001	0.65
		Group × trial type	6.39	<0.05	0.12
		Electrode site × trial type	7.88	<0.05	0.19
	P3	Trial type	41.24	<0.001	0.48
		Group	5.31	<0.05	0.15
		Electrode site	7.19	<0.05	0.19
		Group × trial type	12.93	=0.001	0.15
Latency	N2	Group	6.83	<0.05	0.18
		Electrode site	19.85	<0.001	0.39
	P3	Electrode site × trial type	5.12	<0.05	0.14

**Notes.**

Only significant main effects and interactions are reported in the table.

For the N2 amplitude, a main effect of trial type was identified, indicating that the no-go stimuli (mean [SE], −2.88 [0.76] µV) evoked a larger N2 amplitude than did the go stimuli (mean [SE], −0.77 [0.65] µV). A significant interaction between trial type and electrode site was found, revealing that the increase for the no-go stimuli was weaker in Cz (difference = 1.66 µV) than in Fz (difference = 2.44 µV) or FCz (difference = 2.24 µV). In addition, we determined that there was a significant interaction of group and trial type. A simple effects analysis showed that table tennis athletes elicited a larger N2 amplitude in the no-go trials (mean [SE], −3.57 [1.09] µV) than in the go trials (mean [SE], −0.05 [0.94] µV) (*F*_(1,31)_ = 19.46, *p* < 0.001, *η*^2^ = 0.39). In the non-athlete group, there was no difference between the no-go (mean [SE], −2.20 [1.06] µV) and the go trials (mean [SE], −1.49 [0.91] µV).

Regarding the N2 latency, the main effect of group showed that the latency of the non-athletes (mean [SE], 316.78 [6.26] ms) was longer than that of the table tennis athletes (mean [SE], 293.31 [6.45] ms).

The results for the P3 amplitude showed there was a significant difference between the table tennis athlete group (mean [SE], 20.14 [1.36] µV) and the non-athlete group (mean [SE], 15.76 [1.32] µV). A main effect of trial type was also identified, indicating that the no-go trials (mean [SE], 19.78 [0.98] µV) evoked a larger P3 amplitude than the go trials (mean [SE], 16.13 [1.00] µV). Importantly, an interaction of group and trial type was also identified. Simple effects analyses showed that the table tennis athletes (mean [SE], 22.98 [1.41] µV) elicited a larger P3 amplitude than the non-athletes (mean [SE], 16.57 [1.37] µV) for the no-go stimuli (*F*_(1,31)_ = 10.69, *p* < 0.05, *η*^2^ = 0.26); however, a group difference was not found for the go stimuli (*F*_(1,31)_ = 1.36, *p* > 0.05). Furthermore, a simple effects analysis showed that table tennis athletes elicited a larger P3 amplitude in the no-go trials than in the go trials (*F*_(1,31)_ = 48.70, *p* < 0.001, *η*^2^ = 0.61); non-athletes also elicited a larger P3 amplitude in the no-go trials than in the go trials (*F*_(1,31)_ = 4.12, *p*= 0.05, *η*^2^ = 0.12).

For the P3 latency, only an interaction of trial type and electrode site was found.

#### Strongly masked condition

**Figure 4:**
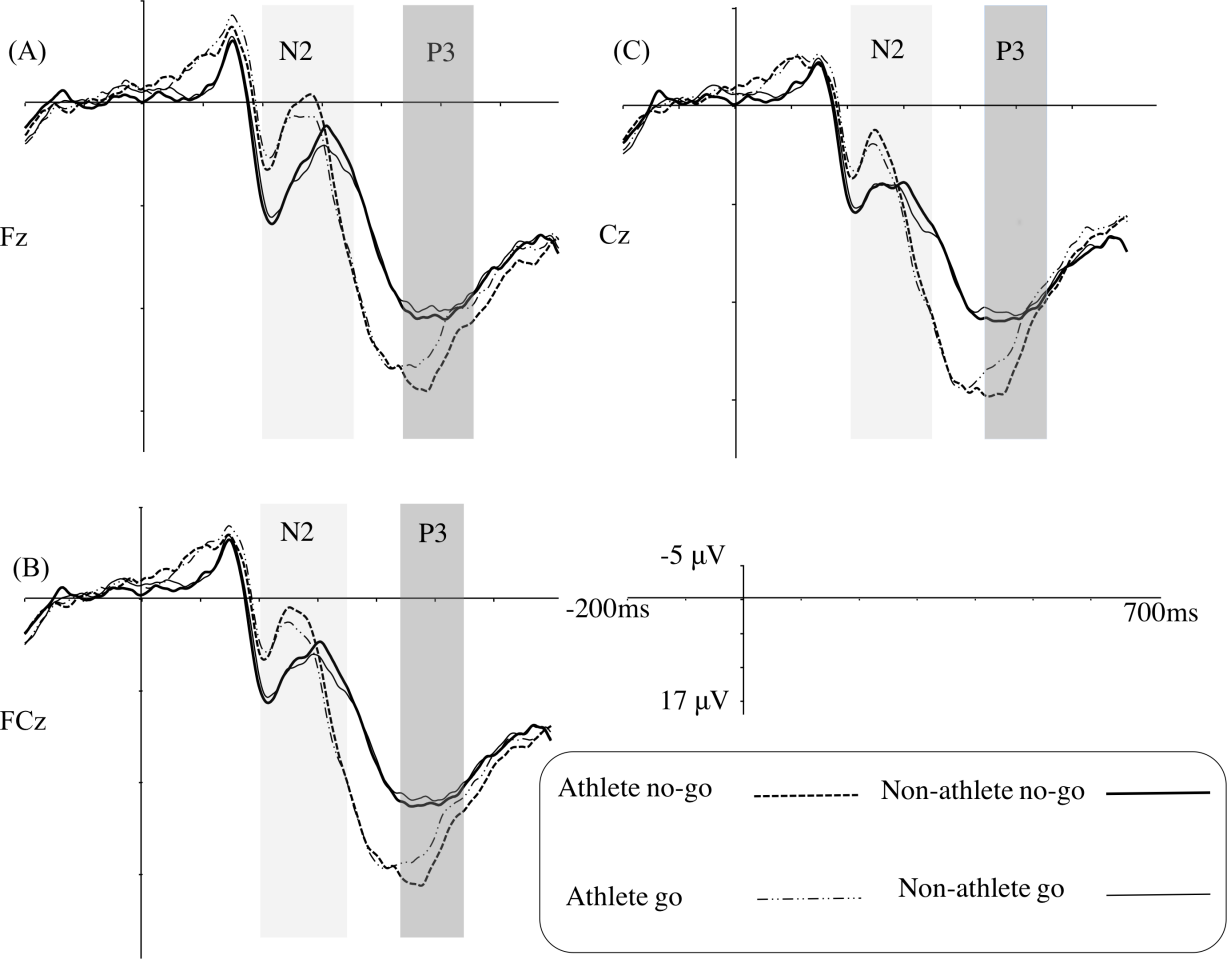
Grand average ERPs in the strongly masked condition for the go and no-go trials in the table tennis athlete and non-athlete groups for each electrode site (A–C represents Fz, FCz, and Cz, respectively).

Figure 4 illustrates the grand average ERPs at Fz, FCz, and Cz for each group in the strongly masked condition, and Table 4 gives the ERP statistical analysis results.

 Only the main effect of electrode site was identified as being significant for the N2 amplitude.

For the N2 latency, the results indicated a main effect of group, revealing that the table tennis athletes (mean [SE], 260.85 [7.28] ms) had a shorter latency than the non-athletes (mean [SE], 293.35 [7.06] ms).

The results of the P3 amplitude showed a main effect of trial type, revealing that the no-go trials (mean [SE], 14.38 [0.83] µV) evoked a larger P3 amplitude than the go trials (mean [SE], 13.29 [0.84] µV). A significant interaction of trial type and group, indicated that the table tennis athletes (mean [SE], 16.26 [1.19] µV) had a larger P3 amplitude than the non-athletes (mean [SE], 12.50 µV [1.16] µV) in the no-go trials (*F*_(1,31)_ = 5.12, *p* < 0.05, *η*^2^ = 0.14); however, a group difference was not found in the go trials. Furthermore, a simple effect analysis showed that table tennis athletes elicited a larger P3 amplitude for the no-go stimuli (mean [SE], 16.26 [1.19] µV) than for the go stimuli (mean [SE], 14.43 [1.20] µV) (*F*_(1,31)_ = 14.84, *p*= 0.001, *η*^2^ = 0.32). In the non-athlete group, there was no difference between the no-go (mean [SE], 12.50 [1.16] µV) and the go stimuli (mean [SE], 12.15 [1.17] µV).

No significant main effects or interaction effects were identified for the P3 latency.

**Table 4:** Statistical analysis results of the electrophysiological data in the strongly masked condition.

**Variable**	**Component**	**Effect**	***F***	***p***	*η*^2^
Amplitude	N2	Electrode site	20.77	<0.001	0.40
	P3	Electrode site	6.48	<0.05	0.17
		Trial type	10.79	<0.05	0.23
		Group × trial type	5.04	<0.05	0.11
Latency	N2	Group	10.27	<0.05	0.25
		Electrode site	15.50	<0.001	0.32

**Notes.**

Only significant main effects and interactions are reported in the table.

## Discussion

Using the masked go/no-go task, we compared the differences in behavioral responses and ERP components during conscious and unconscious response inhibition between table tennis athletes and non-athletes. Our main findings were that table tennis athletes and non-athletes were equally accurate but table tennis athletes had shorter RTs in the weakly masked go trials and displayed longer RT-slowing in the strongly masked condition than non-athletes. In addition, table tennis athletes elicited shorter N2 latencies in all conditions and larger P3 amplitudes in both the weakly and strongly masked no-go trials than non-athletes. These results indicate that table tennis athletes not only display superior conscious response inhibition but also display superior unconscious response inhibition.

### Conscious response inhibition

At the behavioral level, we determined that RTs in the go trials were significantly shorter among the table tennis athletes than among the non-athletes, whereas response accuracy was not different between the groups. These findings are similar to previous results (Chavan et al., 2017; Di Russo et al., 2006; Nakamoto & Mori, 2008b) and underline that athletes from open-skill sports have increased conscious response inhibition speed. Chavan et al. (2017) pointed out that faster conscious response inhibition should manifest itself as an improvement in response speed during the go/no-go task without a concomitant increase in false alarms (i.e., increase in error rate of no-go trials), because an improvement in only the response speed would lead to an increase in false alarms via a speed–accuracy trade-off mechanism. Our findings satisfy this requirement.

At the neural level, there was no group difference for the N2 amplitudes in the no-go trials, a result consistent with a prior study (Nakamoto & Mori, 2008a). We replicated previous results showing that athletes had significantly shorter N2 latencies than non-athletes, regardless of the trial type (Taddei et al., 2012). Previous studies have indicated that in the go/no-go task, the processing of go stimuli provides a mental template for the subsequent visual stimuli; when the no-go stimuli are compared with the mental template, the comparison leads to a mismatch that may elicit conflict monitoring, which is reflected in the amplitude and/or latency of no-go N2 (Ma et al., 2015). Although the amplitude of the no-go N2 is an important index of conflict monitoring, the latency is also important because the latency of no-go N2 indicates the processing speed of conflict monitoring (Gajewski, Stoerig & Falkenstein, 2008). The shorter no-go N2 latency for table tennis athletes indicates that these athletes are quicker than non-athletes in conflict monitoring processes. Contrary to our hypothesis, we only identified a no-go N2 effect (i.e., N2 amplitude is larger in the no-go trials than in the go trials) among the table tennis athletes. Although there was no significant difference in the go N2 amplitudes between the two groups, the figure of grand average ERPs (Fig. 3) showed that non-athletes displayed a tendency for larger go N2 amplitudes than table tennis athletes. According to current conflict monitoring theories, increasing the perceptual overlap between stimuli that hint at frequent and infrequent responses should increase N2 amplitudes (Azizian et al., 2006; Nieuwenhuis, Yeung & Cohen, 2004). Thus, if a go stimulus is similar to a no-go stimulus, the go stimulus may also elicit a conflict because its presentation may cue the wrong response (i.e., response inhibition). Although this interpretation is plausible, an alternative explanation may be that non-athletes did perceive the diamond and square shapes to be similar owing to the short presentation duration of the go/no-go stimulus (17 ms) and the subsequent annulus presentation, and this perceptual overlap may cause the go stimulus to elicit a conflict; thus, the no-go N2 effect disappears in the non-athlete group.

A typical no-go P3 effect (i.e., P3 amplitude is larger in the no-go trials than in the go trials) for the go/no-go task was also identified in our study. Importantly, we replicated previous studies showing that athletes elicited larger no-go P3 amplitudes than non-athletes (Di Russo et al., 2006; Nakamoto & Mori, 2008a; Taddei et al., 2012). The no-go P3 is always considered an index of conscious response inhibition (Falkenstein, Hoormann & Hohnsbein, 1999; Smith, Johnstone & Barry, 2008), and previous studies have argued that an increased no-go P3 amplitude indicates an increased amount of resources (Polich, 2007; Taddei et al., 2012) or a higher conscious response inhibition ability (Nakamoto & Mori, 2008a). Taddei et al. (2012) suggested that the large amplitude of the no-go P3 displayed in fencers may reflect the costs to fencers of increasing the go response speed. To increase the go stimulus response speed, fencers may use a strategy of preparing motor responses to all stimuli, and then when a no-go stimulus is detected, further action is inhibited. Thus, a fencer would require a larger amount of resources for inhibition. In line with Taddei’s hypothesis, large N2 amplitudes in table tennis athletes should also be demonstrated; however, we did not find a main effect of group between our athletes and non-athletes for N2 amplitude. Thus, we suggest that the increased no-go P3 amplitude reflects greater conscious response inhibition ability. Although the predominant assumption is that the no-go P3 amplitude is directly associated with the suppression of overt motor responses, it is usually argued that the no-go P3 amplitude may correspond to the evaluation of inhibitory performance (Huster et al., 2013). Therefore, in our study, the group difference for the no-go P3 amplitude may reflect greater activity in the neural substrates of inhibitory performance evaluation in table tennis athletes than in non-athletes. Fully elucidating the nature of the no-go P3 amplitude will require further experimentation. We used a task without a sports-specific design to estimate conscious response inhibition, and our results were in agreement with a previous study that used sports-specific tasks (Nakamoto & Mori, 2008a; Nakamoto & Mori, 2008b). This indicates that table tennis athletes have a greater ability for conscious response inhibition in general cognitive tasks (Wang et al., 2013).

### Unconscious response inhibition

The main aim of our study was to examine on the differences in unconscious response inhibition between table tennis athletes and non-athletes. At the behavioral level, we replicated the results from previous studies (Wokke et al., 2011; Xu et al., 2015). The main effect of trial type indicates the existence of unconscious response inhibition in both groups. Most importantly, however, we also found longer RT-slowing in table tennis athletes than in non-athletes. Because RT-slowing is a critical index for unconscious response inhibition (Van Gaal et al., 2010; Xu et al., 2015), this result indicates that table tennis athletes display superior unconscious response inhibition at the behavioral level.

At the neural level, we determined that table tennis athletes had shorter N2 latencies than non-athletes, regardless of the trial type. Hence, this suggests that table tennis athletes display faster processing of conflict monitoring, also at the unconsciousness level. However, we did not identify the no-go N2 effect in either group. Van Gaal et al. (2008) conducted a masked go/no-go task which masking technique was similar to our task (i.e., metacontrast masking technique) to explore the existence of unconscious response inhibition and did not identify the no-go N2 effect either. However another study using a masked stop-signal task, which involved sandwich masking technique to explore the existence of unconscious response inhibition, did uncover a significant no-go N2 effect (Van Gaal et al., 2011). Therefore, the no-go N2 effect for unconsciousness may be relatively unstable with its appearance being dependent on the exact masking technique. This needs further scrutiny when addressing unconscious response inhibition in athletes.

In agreement with previous studies (Van Gaal et al., 2011; Van Gaal et al., 2008; Wokke et al., 2011), the unconscious no-go trials elicited a larger P3 amplitude compared with the unconscious go trials (i.e., no-go P3 effect). More importantly, similar to the results for conscious response inhibition, the athletes displayed significantly larger no-go P3 amplitudes than the non-athletes, indicating that the superiority of unconscious response inhibition among table tennis athletes could also be observed at the neural level. Our results are similar to previous results (Van Gaal et al., 2011; Van Gaal et al., 2008; Van Gaal et al., 2010), in that there were similar patterns of neural activity for both conscious and unconscious response inhibition between the groups and that neural activity for unconsciousness is weaker than that for consciousness in both groups. This result is consistent with the idea that both unconscious and conscious no-go stimuli trigger basic inhibition mechanisms.

In sum, the present study showed that table tennis athletes’ not only display superior conscious response inhibition but also display superior unconscious response inhibition. Prior studies have pointed out that athletes from open-skill sports have to process information in a rapidly changing and unpredictable environment, which might lead to superiority in interceptive actions, hand-eye coordination and perception-action or inhibition of inappropriate movements or response selection (Wang et al., 2013; Lees, 2003). Therefore, athletes from open skill sports are likely to have improved conscious response inhibition due to long-term response inhibition experience and perhaps dedicated training (open-skill sport training). Interestingly, previous studies related to response control training have indicated that short- or medium-term training of response control changes the gray and white matter in the inferior frontal gyrus (e.g., Chavan et al., 2015), and a recent research using elite fencers has suggested that long-term response control training (i.e., fence training) changes the white matter microstructure of the fronto-basal response control network (Chavan et al., 2017). It appears reasonable therefore to suggest that the changes in the structure of the fronto-basal response control network by long-term response control training in athletes of open skills sports, including table tennis athletes will affect the feedforward sweep. Because unconscious and conscious stimuli have been shown to travel along similar processing routes during feedforward sweep (even in the prefrontal cortex) (Van Gaal et al., 2008), this experience and training not only results in superior conscious response inhibition but also in superior unconscious response inhibition.

The ERPs recorded in the present study were unexpectedly large. One reason for this may have been our use of the masked go/no-go task because a previous study using this task also showed large ERP amplitudes (Xu et al., 2015). However, another likely reason could be our filter parameters. We used only a low-pass filter to screen the EEG data. A previous study that also used only a low-pass filter also showed large ERP amplitudes (Ma et al., 2015); even in the masked go/no-go task, the ERP amplitudes were markedly smaller when a high-pass filter of 0.5 Hz and a low-pass filter of 30 Hz were used to screen EEG data (Wokke et al., 2011).

Our study has another limitation that should be acknowledged: we used a cross-sectional design. Although this approach can explore the differences in unconscious response inhibition between table tennis athletes and non-athletes, it cannot rule out the possibility that the differences are caused by genetic factors rather than long-term practice. Future research should include longitudinal studies to explore this possibility.

## Conclusion

The present study results demonstrated that table tennis athletes have advantages in both conscious and unconscious response inhibition compared to non-athletes.

##  Supplemental Information

10.7717/peerj.5548/supp-1 Data S1 Raw data

10.7717/peerj.5548/supp-2 Supplemental Information 1 The International Physical Activity Questionnaire

## Additional Information and Declarations

### Data Availability

The following information was supplied regarding data availability: The raw data is provided in a Supplemental File and at Figshare: You, Yihong; Ma, Yiming; Ji, Zhiguang; Meng, Fanying; Li, Anmin; Zhang, Chunhua (2018): Unconscious response inhibition differences between table tennis athletes and non-athletes. figshare. Dataset. https://doi.org/10.6084/m9.figshare.5782941.v1.
