# Supplementary material for: Unconscious response inhibition differences between table tennis athletes and non-athletes
Source: PeerJ. 2018 Sep 7;6:e5548. doi: 10.7717/peerj.5548 (PMC6130236; doi:10.7717/peerj.5548)
Supplement: Supplemental Information 1 [file peerj-06-5548-s002.doc]

# The International Physical Activity Questionnaire

**Gender_________ Age_________ Code_________**

**Height________(m) Weight________(kg)**

We are interested in the kinds of physical activities that people do as part of their everyday lives.

Please answer each question. Please think about the activities you do at work, as part of your house, to get from place to place, and in your spare time for recreation, exercise or sport.

**Think about only those vigorous physical activities that you did for at least 10 minutes at a time. Vigorous physical activity refer to activities that take hard physical effort and make you breathe much harder than normal.**

1. During the last 7 days, on how many days did you do vigorous physical activities like aerobics, fast bicycling？

days per week

**No vigorous activity**
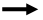
**skip to question 3**

1. How much time did you usually spend on one of those days doing vigorous physical activities?

hours per day

minutes per day

________ not known

**Think about only those moderate physical activities that you did for at least 10 minutes at a time. Moderate physical activity refer to activities that take moderate physical effort and make you breathe somewhat harder than normal.**

1. During the last 7 days, on how many days did you do moderate physical activities, like carrying light loads, bicycling, tennis doubles？Not including walking**.**

days per week

**No moderate activity**
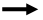
**skip to question 5**

1. How much time did you usually spend on one of those days doing moderate physical activities：

hours per day

minutes per day

________ not known

**Think about your walking that you did for at least 10 minutes at a time, including at work, at home, to get from place to place and so on.**

1. During the last 7 days, on how many days did you walk for at least 10 minutes？

days per week

**No walking**
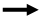
 **skip to question 7**

1. How much time did you usually spend on one of those days walking？

hours per day

minutes per day

________ not known

**The last questions are about the time you spend sitting while at work, at home, while doing course work and during leisure time. This may include time spent sitting at a desk, visiting friends, reading or sitting or lying down to watch television.**

1. During the last 7 days, how much time did you usually spend sitting on a weekday?

hours per day

minutes per day
